# PD10 Cost Effectiveness Analysis Of Etranacogene Dezaparvovec Versus Factor IX Prophylaxis In Adult Men With Hemophilia B From Brazil

**DOI:** 10.1017/S0266462324002782

**Published:** 2025-01-07

**Authors:** Ana Clara Silva Mendes, Ricardo Mesquita Camelo, Juliana Alvares-Teodoro

## Abstract

**Introduction:**

Hemophilia B (HB) is a rare bleeding disorder caused by clotting factor IX (FIX) deficiency. Regular FIX replacement (prophylaxis) is the mainstream treatment for preventing bleeding in people with HB. Etranacogene dezaparvovec, also known as AMT-061, is a new gene therapy approved in many countries for people with HB. This study evaluated the cost effectiveness of conventional FIX prophylaxis compared with AMT-061 for people with HB.

**Methods:**

A cost-effectiveness decision tree was constructed based on current literature and national guidelines on HB treatment. The model was based on a Brazilian Ministry of Health perspective, assumed that people with severe HB were 18 years or older, and used a time horizon of three years. The Brazilian Ministry of Health distributes plasma-derived FIX (pdFIX) for prophylaxis. The cost of pdFIX was obtained from data on public purchases and AMT-061 cost was based on the price of a currently approved gene therapy in Brazil. Sensitivity analyses were performed to assess uncertainty and to identify which variables most affected the incremental costs.

**Results:**

The cost of prophylaxis with pdFIX for three years was BRL233,309 (USD41,236) with an annualized bleeding rate (ABR) of 10.95, while the cost of gene therapy for three years was BRL10,379,041 (USD1,834,466) with an ABR of 2.86. This resulted in an incremental cost-effectiveness ratio (ICER) of BRL1,253,674 (USD221,583) per bleeding episode. Compared with pdFIX, AMT-061 was not cost effective since there was a threshold of BRL30,000 (USD5,302) per bleeding episode avoided. In the deterministic sensitivity analysis, treatment time had a major impact on the ICER, followed by AMT-061 cost.

**Conclusions:**

In this model, AMT-061 was not cost effective compared with pdFIX prophylaxis. In the long term or with a lower AMT-061 cost, gene therapy may be cost effective. AMT-061 demonstrated better clinical results, but its economic viability must be considered in terms of access and sustainability in the Brazilian public healthcare system.

